# Contraception in chronic kidney disease: a best practice position statement by the Kidney and Pregnancy Group of the Italian Society of Nephrology

**DOI:** 10.1007/s40620-020-00717-0

**Published:** 2020-03-12

**Authors:** Rossella Attini, Gianfranca Cabiddu, Benedetta Montersino, Linda Gammaro, Giuseppe Gernone, Gabriella Moroni, Domenico Santoro, Donatella Spotti, Bianca Masturzo, Isabella Bianca Gazzani, Guido Menato, Valentina Donvito, Anna Maria Paoletti, Giorgina Barbara Piccoli

**Affiliations:** 1 Department of Obstetrics and Gynecology, Città della Salute e della Scienza, Ospedale Sant’Anna, Turin, Italy; 2 Nephrology, Azienda Ospedaliera Brotzu, Cagliari, Italy; 3 Nephrology Ospedale Fracastoro San Bonifacio, San Bonifacio, Italy; 4 Nephrology, Ospedale Santa Maria Degli Angeli, Putignano, Italy; 5 Nephrology, Fondazione Ca’ Granda Ospedale Maggiore, Milan, Italy; 6 Nephrology and Dialysis, Azienda Ospedaliera Universitaria “G. Martino”, Messina, Italy; 7 Nephrology and Dialysis, IRCCS Ospedale San Raffaele, Milan, Italy; 8 Department of Internal Medicine, Ospedale Sant’Anna, Città della Salute e della Scienza, Turin, Italy; 9 Department of Surgical Sciences, Obstetrics and Gynecology, University Hospital of Cagliari, Cagliari, Italy; 10 Department of Clinical and Biological Sciences, Università di Torino, Turin, Italy; 11 Nephrology and Dialysis, Centre Hospitalier Le Mans, Le Mans, France

**Keywords:** Contraception, Birth control, Chronic kidney disease, Dialysis, Kidney transplantation, Abortion, Hormonal contraceptives, Intrauterine devices, Barrier methods, Emergency contraception

## Abstract

Even though fertility is reduced, conception and delivery are possible in all stages of CKD. While successful planned pregnancies are increasing, an unwanted pregnancy may have long-lasting deleterious effects, hence the importance of birth control, an issue often disregarded in clinical practice. The evidence summarized in this position statement is mainly derived from the overall population, or other patient categories, in the lack of guidelines specifically addressed to CKD. Oestroprogestagents can be used in early, non-proteinuric CKD, excluding SLE and immunologic disorders, at high risk of thromboembolism and hypertension. Conversely, progestin only is generally safe and its main side effect is intramestrual spotting. Non-medicated intrauterine devices are a good alternative; their use needs to be carefully evaluated in patients at a high risk of pelvic infection, even though the degree of risk remains controversial. Barrier methods, relatively efficacious when correctly used, have few risks, and condoms are the only contraceptives that protect against sexually transmitted diseases. Surgical sterilization is rarely used also because of the risks surgery involves; it is not definitely contraindicated, and may be considered in selected cases. Emergency contraception with high-dose progestins or intrauterine devices is not contraindicated but should be avoided whenever possible, even if far preferable to abortion. Surgical abortion is invasive, but experience with medical abortion in CKD is still limited, especially in the late stages of the disease. In summary, personalized contraception is feasible, safe and should be offered to all CKD women of childbearing age who do not want to get pregnant.

## Introduction

Chronic kidney disease (CKD) is usually cited as a cause of reduced fertility, but in fact this simplistic assumption is probably true only for the last stages of the disease, or for some immunologic diseases, of which systemic lupus erythematosus (SLE) can be considered the prototype [1–4].

Almost paradoxically, we have more information on fertility and pregnancy rates in the late stages of kidney disease, mainly because CKD is known and acknowledged, than in the early CKD stages, in which a significant number of women, perhaps even the majority, do not realize they are affected by CKD, or do not consider that this is relevant in determining pregnancy outcomes [1, 5–7].

While several recent studies have been addressed to women with CKD and their desire, still too often frustrated, to give birth, contraception is not a part of the routine work-up for CKD patients, notwithstanding the fact that, albeit with lower rates, pregnancy is possible in all CKD stages, including transplantation and dialysis [1, 2, 8–12].

It is in particular in the late CKD stages that the widespread idea that fertility is sharply reduced may cause doctors to overlook counselling on contraception, with potentially devastating clinical and psychological effects. Careful planning of pregnancy is vitally important in all disease stages, to avoid complications for mother (for example in the case of active immunologic diseases) and foetus (for example exposure to potentially teratogenic drugs) [13–16].

Some recent reviews have addressed the issue of contraception in CKD; they all acknowledge the lack of detailed, specific evidence deriving from primary studies on CKD women [17–21]. The high heterogeneity of CKD stages, type of disease and treatment is one of the reasons for this lack of evidence or of the use of low-quality evidence, even in the fields most often studied (kidney transplantation, lupus nephropathy and diabetic nephropathy) [20–23].

With this in mind, the Italian Society of Nephrology’s Project Group on Kidney and Pregnancy has undertaken the preparation of the present best practice statement. We will try to summarize the available evidence and provide answers to open questions on contraception in CKD integrating the currently available “best practice” statements on pregnancy in CKD, dialysis, kidney transplantation and follow-up after preeclampsia [24–27].

Our review will first discuss the main modes of contraception available for the general population, and then try to contextualize them in the various stages of CKD, in dialysis and after kidney transplantation, with particular regard to the potential interaction between contraceptives and the pharmacologic treatments most commonly used in CKD.

## World Health Organization (WHO) criteria for election of contraception

Analogously to its classification of drug teratogenicity, the World Health Organization (WHO) provides a general score for supporting contraceptive eligibility in different diseases (Table 1) [28]. The safety of every contraceptive is classified in one of four categories: in categories 1-2 the use of the contraceptives is with no or minimal restriction, in categories 3 and 4, WHO advises avoidance, (the strength of recommendations varies in and motivated exceptions are proposed in category 3).

**Table 1:** Medical Eligibility Criteria (MEC) for contraceptive use (WHO 2015) [28]

MEC categories for contraceptive eligibility	
1	A condition for which there is no restriction for the use of the contraceptive method
2	A condition where the advantages of using the method generally outweigh the theoretical or proven risks
3	A condition where the theoretical or proven risks usually outweigh the advantages of using the method
4	A condition which represents an unacceptable health risk if the contraceptive method is used

*MEC* Medical Eligibility Criteria, *WHO* World Health Organization

The evidence on which WHO criteria were based came from studies on a wide range of diseases and conditions, among them diabetes, hypertension, SLE and kidney transplantation. Chronic kidney disease was, however, not included.

WHO’s Medical Eligibility Criteria (MEC) for contraception are based on two complementary considerations regarding safety: first, the effect the contraceptive has on the disease (worsening or increasing risk); second, the effect the disease has on the efficacy of the contraceptive method.

WHO’s most recent classification considers the following contraceptive methods:

- Combined hormonal contraceptives (CHC)
- low dose (≤ 35 mcg Ethinyl Estradiol) combined oral contraceptives (COCs).
- Combined patch (P).
- Combined vaginal rings (CVR).
- Combined injectable contraceptives (CICs) *.
- Progestin-only pills (POPs).
- Depot-medroxyprogesterone acetate (DMPA)*.
- Norethisterone enanthate (NET-EN)*.
- Levonorgestrel (LNG)* and Etonogestrel (ETG) implants.
- Emergency contraceptive pills (ECPs).
- Copper-bearing intrauterine devices (Cu-IUDs).
- Levonorgestrel-releasing IUDs (LNG-IUDs).
- Copper-IUDs for emergency contraception (E-IUD).
- Progesterone-releasing vaginal rings (PVR)*.
- Barrier methods (BARR).
- Fertility awareness-based methods (FAB).
- Lactational amenorrhoea method (LAM).
- Coitus interruptus (CI).
- Female and male sterilization (STER).

*Not available in Italy.

In our review we use WHO’s categories of contraceptive methods and their specific Medical Eligibility Criteria (MEC). Obviously, as is emphasized in the MEC, counselling must be personalized and consider individual characteristics and risk factors; those generally listed include age, breastfeeding status, dyslipidaemia, severe cardiovascular disease, migraine, severe liver disease, concomitant therapies and body mass index. Kidney diseases are not specifically considered, hence the need for the present best practice statement.

## Efficacy of contraceptive methods

The efficacy of a contraceptive method is conventionally measured by the Pearl Index, i.e. the number of pregnancies in 100 women using the method for 1 year (1300 periods). The lower the number, the more effective the method is. It should be noted that to some extent the Pearl Index depends on the user population’s characteristics, training in correct use of the method and the fertility of the couples included.

A recent US review proposes a classification of contraceptive effectiveness stratified for “optimal use” or “typical use” (taking inaccuracies into account); it also analyses adherence to methods after 1 year of use. Apart from surgical methods (which are obviously definitive), compliance is high for implants and IUDs, which can remain in place for 3–5 years, but adherence to combined hormonal contraceptives was as low as 67%. Barrier methods are quite effective with “perfect” use, but typical use produces a high percentage of unintended pregnancies; the same applies to withdrawal and ovulation methods (Table 2) [29].

**Table 2:** Percentage of failure of the main contraceptive methods and percentage of women continuing the method at 1 year. Adapted from (Trussell 2007) [29]

Method	% of women experiencing an unintended pregnancy within the first year of use		% of women continuing use at 1 year
	Typical use	Perfect use	
No method	85	85	–
Non-pharmacological methods			
Female condom	21	5	41
Withdrawal	20	4	46
Diaphragm	17	16	57
Ovulation method	23	3	
Male condom	13	2	43
Copper IUD	0.8	0.6	78
Pharmacological methods			
COCs and POPs	7	0.3	67
Patch	7	0.3	67
Vaginal ring	7	0.3	67
Injectable	4	0.2	56
LNG-IUD	0.1–0.2	0.1–0.2	80
Implant	0.1	0.1	89
Surgical methods			
Tubal occlusion	0.5	0.5	100
Vasectomy	0.15	0.1	100

*IUD* intrauterine device, *COC* combined oral contraceptive, *POP* progestin-only pill, *LNG-IUD* levonorgestrel intrauterine device

There are also differences between countries regarding availability and choice of contraceptives. For example, in Italy CICs are not available and implants are rarely used. Male sterilization is rare, as in the rest of the world; male condom use and withdrawal are widely used, with consequences linked to poor efficacy (Table 3) [30].

**Table 3:** Percentage of couples in fertile age that chose a specific contraceptive method in 2018. Adapted from the Population Reference Bureau, 2019 [30]

Method	World	Europe	Italy	Developing countries
All methods	62	70	65.1	54
Non-pharmacological methods				
IUD	13	11	4.8	13
Condom	8	21	20.9	6
Withdrawal	4	2	17.5	3
Pharmacological methods				
Oral contraceptives	9	20	20.3	8
Surgical methods				
Male sterilization	–	–	–	–
Female sterilization	18	22	5.8	20

*IUD* intrauterine device

## Design of the best practice position statement

The present best statement has been designed, in collaboration with obstetric teams with experience in the management of kidney patients, to support clinical nephrologists with practical indications on the most commonly used birth control methods and treatments, with the final aim of giving nephrologists the tools they need to actively participate in the choice of a contraceptive agent, in keeping with obstetric indications and patient preferences.

It is organized according to type and efficacy of contraceptive agents, trying to identify, for each of them, the limits and the specific indications or contraindications in different categories of CKD patients. The evidence related to CKD is low, and the indications and suggestions given are based on similar diseases (immunologic diseases), on CKD complications (hypertension) or on the presence of related diseases, such as diabetes. Within these limits, we have tried to summarize a counselling policy suitable for women in different phases of CKD, dialysis and transplantation.

## Combined hormonal contraceptives (aestro-progestinic agents agents)

- Combined hormonal contraceptives (CHCs) are widely used and highly effective, but the cardiovascular and thrombotic risks are high and this can be of particular relevance for CKD patients (grade of evidence: high for the general population, low for the specific categories related to CKD patients).
- There is no absolute contraindication for CHC use in CKD patients without hypertension or immunologic diseases or those taking immunosuppressive drugs (grade of evidence: low for the specific categories related to CKD patients).
- No correlation between CHC and neoplastic risk has been established. An increase in incidence of breast and cervical cancer is debated; likewise, the protective effect on ovarian, endometrial and colorectal cancer is controversial (grade of evidence: debated in relation to the general population, no studies on CKD patients).
- CHC can induce or worsen albuminuria; this side effect is usually reversible at discontinuation in patients without CKD. The pathogenesis is not known but seems to be independent from blood pressure (grade of evidence: high for the general population, no studies on CKD patients)
- The use of a patch is associated with higher circulating hormonal levels, with consequently higher risk of adverse effects (grade of evidence: high for the general population, low for the specific categories related to CKD patients).
- Vaginitis and leucorrhoea are more frequent with a vaginal ring and a ring may favour urinary tract infections (grade of evidence: high for the general population, low for the specific categories related to CKD patients).
- Alternatives should be systematically sought in advanced and progressive CKD, immunologic diseases, and complicated kidney transplantation; the presence of proteinuria and hypertension increases the risk of adverse effects (grade of evidence: low for the specific categories related to CKD patients, but supported by shared clinical views).

Combined hormonal contraceptives are contraceptives that contain different types of oestrogen and progestin, combined in different dosages. They are the first, and still the most widely used contraceptives, colloquially referred to as “the pill”. They are highly effective (Pearl Index < 1 with perfect use, see Table 2). Combined hormonal contraceptives exist in four forms: oral contraceptives (COCs), transdermal patch (P), transvaginal ring (CVR) and combined injectable contraceptives (CICs).

Besides being used for birth control, combined hormonal oral contraceptives are routinely prescribed to non-CKD patients for dysmenorrhea (painful menstruation), functional ovarian cysts, hypermenorrhea (abundant menstruation), polymenorrhea (frequent menstruation), endometriosis or acne.

The Medical Eligibility Criteria take very few nephropathies into account: diabetic nephropathy in category 3 or 4 (depending on severity), while diabetes without vascular disease is included in category 2; when normotensive, well controlled and in remission, SLE is also included in category 2 but the presence of antiphospholipid antibodies warrants its inclusion in category 4 (absolute contraindication). Kidney transplantation is categorized in more than one way (category 2 if “non- complicated”, up to 4 in the presence of organ failure or acute/chronic rejection). Hypertension, which often coexists in kidney disease, is in category 3, even when moderate and controlled by therapy. [28]

Combined oral contraceptives (COCs) or “the pill”, are one of the most effective contraceptive methods (failure rate with perfect use: 0.3%, Table 2) and are widely used throughout the world. Birth control pills contain different types of two hormonal components, progestin and oestrogen, combined in different dosages. COCs inhibit the release of GnRH, thus blocking the release of the hypophysis hormones that stimulate ovulation, alter the endometrium and cause thickening of the cervical mucus. Birth control pills must be taken for between 21 and 24 days per month, with 4–7 days of pause between cycles, in which a suspension bleeding appears. Some COCs have the same dosage during the entire month (monophasic pills), others have different dosages (multiphasic pills), in order to better mimic natural hormonal fluctuations. The oestrogen typically associated with progestin is Ethinyl Estradiol (EE), with a dosage that varies from 10 to 35 mcg/day (“low-dosage oestrogens” < 35 mcg/day). New oestrogen-derivatives have recently been introduced (Estrogen Valerate, Estradiol) to minimize the oestrogen-related adverse effects of taking birth control pills, since they induce less liver protein synthesis (e.g. sex-hormone binding globulin, angiotensinogen and coagulation factors) (Table 4).

**Table 4:** Main non-thromboembolic side effects of CHCs

Target	Complication
Gastrointestinal	Nausea
Dermatological	Acne
Neurobehavioural	Decreased libido
	Depressed mood
	Headache
	Increased appetite
Gynecological	Breast tenderness
	Breakthrough bleeding
	Increased vaginal discharge

The first generation of contraceptive pills, developed in the 1960s, used a high concentration of oestrogen with progestin with androgenic activity; the second generation of hormonal contraceptives combined lower levels of oestrogens with various testosterone-derived progestins characterized by low but significant androgenic activity; the most widely used progestin in this category is Levonorgestrel (LNG). COCs in combination with Levonorgestrel have the lowest thromboembolic risk (Tables 5, 6) [31], but their androgenic effects must be considered before prescribing them to women with acne or hirsutism. Since the 1990s, further combined hormonal contraceptives have been developed, with different progestins; these are sometimes referred to as third-generation (those containing Desogestrel, Gestodene or Norgestimate) and fourth-generation contraceptives (those containing Norelgestromine, Etonogestrel, Drospirenone or Dienogest). The classification in different “generations”, however, is not standardized, and may differ between publications, thus making it advisable to refer to the specific contents and not merely to a “generation”.

**Table 5:** Risk of developing deep venous thrombosis in per year of use. Modified from the European Medicines Agency, 2014 [31]

Hormonal contraceptive use Combined hormonal contraceptives (CHCs)	Cases of deep venous thrombosis
No CHC (and not pregnant)	2/10,000 women
CHC containing Levonorgestrel, Norethisterone or Norgestimate	5–7/10,000 women
CHC containing Etonogestrel or Norelgestromin	6–12/10,000 women
CHC containing Drospirenone, Gestodene or Desogestrel	9–12/10,000 women
CHC containing Chlormadinone, Dienogest or Nomegestrol	Not yet known^a^

*CHC* combined hormonal contraceptive

^a^Further studies are ongoing or planned to collect more data

**Table 6:** Odds ratios comparing the risk of non-fatal venous thromboembolism in users of different contraceptives. Adapted from MEGA study [33]

Type of progestin	OR	95% CI
Levonorgestrel	3.6	2.9–4.6
Gestodene	5.6	3.7–8.4
Norgestimate	5.9	1.7–21.0
Drospirenone	6.3	2.9–13.7
Ciproterone acetate	6.8	4.7–10.0
Desogestrel	7.3	5.3–10.0

*OR* odds ratio, *CI* confidence interval

The transdermal CHC or combined patch (P) is a 4.6-cm^2^ patch that must be replaced every week, for three consecutive weeks, followed by a patch-free week. The active principles contained in a single patch are Ethinyl Estradiol (6 mg) and Norelgestromin (600 mcg), releasing on average 34 mcg/day of Ethinyl Estradiol and 203 mcg/day of Norelgestromin. Efficacy and cycle control are similar to those of COCs, but the patch has the advantage of avoiding first-pass effect in the liver. The circulating levels of oestrogen are usually higher in transdermal than in oral CHCs.

The combined vaginal ring (CVR), manufactured from ethylene-co-vinyl acetate, is a flexible atoxic ring measuring 5.4 cm in diameter that contains Etonogestrel (11.7 mg) and Ethinyl Estradiol (2.7 mg), releasing on average 120 mcg/day of Etonogestrel and 15 mcg/day of Ethinyl Estradiol. The ring is kept in the vagina for 3 consecutive weeks, and is replaced after 1 ring-free week. Efficacy and control of the cycle are optimal, and the principles are not subject to first pass effect in the liver. Moreover, the oestrogen dose is very low.

The recently developed injectable CHCs (combined injectable contraceptives) contain either 25 mg of Depot-medroxyprogesterone acetate and 5 mg of Estradiol Cipion or 50 mg of Norethisterone Enanthate and 5 mg of Estradiol Valerate. Combined injectable contraceptives have good pharmacokinetics and are less subject to individual variations, but can cause spotting and bleeding. Combined injectable contraceptives are currently not available in Italy and are mostly used in developing countries, because the therapy is easy to manage (one injection per month) and inexpensive.

## Risks and side effects of CHCs

In spite of their high efficacy, CHCs are not devoid of side effects. Table 4 reports their main non-thromboembolic side effects.

Thromboembolic and cardiovascular events are rare, although potentially severe; the risk of venous thromboembolism varies between CHCs, depending on the amount of oestrogen and type of progestin they contain, ranging from 5 to 12 cases per 10,000 women, a two- to five-fold increase with respect to the usual incidence of 2 per 10,000 in women not using CHCs (Tables 5, 6) [31]. Overall, the risk is higher in the first year of use and is correlated with oestrogen dose. A meta-analysis published in 2018 analysed the risk of venous thrombosis associated with different types of combined oral contraceptives: the risk was lowest on COCs containing Levonorgestrel, followed by Gestodene and Cyproterone and highest on those containing Desogestrel (according to Oedingen, RR 1.46; 95% CI 1.33–1.59 [32]). However, many studies lack information on potential confounders such as family history, body mass index, smoking and, most importantly, duration of use [32–36].

The association between the use of oral contraceptives and hypertension has been known since 1967, but the pathogenesis is still not fully clear. Genetic predisposition, duration of treatment, age and obesity are probably contributing factors. Oestrogens stimulate the hepatic synthesis of angiotensinogen, and activate the renin-aldosterone system; renal and peripheral hemodynamic alterations have been reported. Progestins can also increase blood pressure, albeit less significantly, probably via increased sodium retention; the effect seems to be of shorter duration, leading to the conclusion that progestin-only agents are less dangerous from this point of view [37–40].

A large body of evidence supports an increased risk of developing or worsening of albuminuria (OR 1.90; CI 1.23–2.93, according to Monster 2001 [43]). The increase is usually modest and reversible after discontinuation of CHCs, but must be taken into consideration when a CHC is prescribed to a CKD patient. The increase in urinary albumin excretion is modulated by age (increasing in older women) and type of oestrogen; it tends to be higher on second- or third-generation CHCs. The pathogenesis is not fully known, but may be related to a haemodynamic effect. This side effect underlines the fact that CHCs’ long-term effects on kidney function are not fully understood. Although there is currently no evidence of a CHC-related predisposition to renal disease, some authors suggest that CHCs may represent a “first hit” in the development of CKD. In case of prescription of CHCs to patients with a kidney ailment, it is advisable to carry out periodic controls of proteinuria [40–45].

Drospirenone and the other fourth-generation progestins sometimes increase potassium levels because of their anti-mineralocorticoid effects, and should be used with care in patients with electrolyte disturbances [46].

The evidence of CHCs posing a cancer risk is controversial, as studies are highly heterogeneous in terms of population characteristics, type of the contraceptive, and duration of assumption. Furthermore, the long-term studies we have regard mainly first-generation, high-dosage pills, and the long-term effects of the more recent formulations are not yet known.

Several epidemiological studies and a systematic review published in 2003 reported an association between HPV-related cervical cancer and use of COCs; the data was not confirmed in a recent meta-analysis, suggesting that this association is related to the fact that condoms are seldom used in patients on CHCs, with a consequent higher incidence of HPV infections [47–56].

An increase in breast cancer is controversial; some data suggest a slight but significant increase in women using COCs (Gierisch 2013: OR 1.08; 95% CI 1.00–1.17 [56]), most frequently in triple-negative forms. The incidence of breast cancer decreases 5–10 years after a woman stops taking birth control pills [49, 56–69].

Conversely, the incidence of some cancers is significantly reduced by oral contraceptive use, regardless of the duration of therapy. This holds true for colorectal cancer (see Gierisch 2013: OR 0.86; CI 0.79–0.95 [56]) and endometrial cancer (see Gierisch 2013: OR 0.57; CI 0.43–0.77 [56]). CHCs are also associated with benign hepatic tumours, including hepatic adenomas or focal nodular hyperplasia, which rarely turn into a malignant hepato-cellular carcinoma. Furthermore, COC users, whether they are without predisposing factors or are carriers of BRCA1/BRCA2, apparently have a reduced risk of ovarian epithelial cancer [49, 56, 57, 70–79].

In summary, CHCs seem to protect against some types of cancers and to increase the risk of developing others. Since quantifying this risk is extremely difficult, the risk balance could be considered neutral and the decision on whether or not to prescribe this type of contraceptive should be based on other considerations. The only exception, according to WHO, is the presence or history of breast cancer.

The side effects of taking CHCs could be expected to be similar in their transdermal, vaginal and oral formulations; however, a Cochrane review published in 2013 [80] showed that patch users have a higher rate of discontinuation than oral contraceptive users, due to adverse effects including breast discomfort, dysmenorrhea, nausea and vomiting, and local irritation. Ring users also reported less nausea, acne, irritability, depression, and emotional problems than COC users. The main complaints recorded with the transvaginal ring include vaginitis (OR 2.48; CI 1.39–4.43 with respect to oral agents [80]) and leucorrhoea (OR 3.21; CI 1.61–6.40 [80]), but less vaginal dryness. These effects may be of particular relevance in immunosuppressed patients or in patients with frequent urinary tract infections [80].

A potential favourable aspect is the fact that transdermal and vaginal preparations do not undergo first pass liver metabolism; therefore, they have a lower risk of potentially dangerous pharmacologic interferences involving the cytochrome P4503A4. This is particularly important in the case of calcineurin inhibitors, metabolized by the same pathway. In fact CHCs and progestin-only contraceptives have an inhibitory effect on P4503A4, thus increasing the concentration of calcineurin inhibitors, in particular Cyclosporine. Conversely, calcineurin inhibitors (in particular Tacrolimus) have an inductive effect on P4503A4, potentially reducing contraceptive efficacy [81–83].

Progestin-only contraceptives (POPs, injectables, implants)

Progestin-only contraceptives represent an effective alternative to CHCs, mainly due to a better cardiovascular profile (grade of evidence: high for the general population, low for the specific categories related to CKD patients).

There is no absolute contraindication for progestin-only contraceptive use in CKD patients (grade of evidence: high for the general population, low for the specific categories related to CKD patients).

Weight gain is a potential side effect (grade of evidence: high for the general population, low for the specific categories related to CKD patients).

Potential interference with liver metabolism should be considered in cases of calcineurin inhibitors (grade of evidence: high for the general population, low for the specific categories related to CKD patients).

Progestin-only contraceptives act through inhibition of the release of GnRH, by modifying the composition of the cervical mucus making it impenetrable, and by reducing endometrial thickness, impairing implantation, and affecting tubal motility. The main advantage of progestin-only contraceptives lies in their minimal (or absent) impact on coagulation and blood pressure, thus making them good alternatives in women in a WHO MEC category 3 or 4 for combined hormonal contraceptives, which is the case for many women with CKD. Furthermore, these agents can be used postpartum and during lactation. However, although usually mild, weight gain can be an important drawback, in particular for obese and overweight patients [29, 84–88].

The contraindications and warnings identified by WHO are generally mild: category 1–2 hypertension; SLE 2, and SLE 3, in the presence of anti-phospholipid antibodies (Tables 7, 8). Kidney transplantation is rated 2, independently from the presence of hypertension, proteinuria or functional reduction. Diabetes-related nephropathy is rated 2 regarding progestin-only pills or implants, and rated 3 with respect to injectable progestins [31]. Several different approaches are available: POPs (progestin-only pillx or “minipillx”); and the depot method, either via intramuscular administration or a subdermal implant.

**Table 7:** WHO indications regarding hypertension and diseases of nephrological interest

Disease	POPs, implants, injectable	Cu-IUD	LNG-IUD
Adequately controlled hypertension	1^a^	1	1
Diabetic nephropaty	2^b^	1	2
SLE	2	1	2
SLE + positive antiphospholipid antibodies	3	1	3
Uncomplicated kidney transplantation	2	2	2
Complicated kidney transplantation	2	2 (continuation)/3 (initiation)	2 (continuation)/3 (initiation)

*WHO* World Health Organization, *SLE* systemic lupus erytematosus, *CHC* combined hormonal contraceptive, *POP* progestin-only pill, *Cu-IUD* copper-bearing intrauterine device, *LNG-IUD* levonorgestrel intrauterine device

^a^Cat.2 implant

^b^Cat. 3 injectable

**Table 8:** Indications on contraception for SLE patients. Adapted from references [28, 95, 106]

	CHCs	POPs	LNG-IUD
SLE			
WHO	2	2	2
EULAR	Can be considered	Not available	Can be offered to all patients
SAMMARITANO	No increased flare in stable patients	No risk flare	No risk flare
SLE + aPL			
WHO	4	3	3
EULAR	controindicated	Carefully weighed against the risk of thrombosis (2B)	Can be offered to all patients
SAMMARITANO	Increased risk thrombosis. AVOID	No risk thrombosis	No risk thrombosis

*CHC* combined hormonal contraceptive, *POP* progestin-only pill, *LNG-IUD* levonorgestrel-intrauterine device, *SLE* systemic lupus erythematosus, *aPL* antiphospholipid antibodies, *WHO* World Health Organization, *EULAR* the European League Against Rheumatism

POP therapy available in Italy consists of Desogestrel (75 mcg/day) that must be taken every day, without interruptions; elsewhere in Europe and in other settings, the most widely used minipills contain Desogestrel (as in Italy) or Norethisterone (350 mcg/day); Norethisterone is the only POP available in the United States. The failure rate with perfect use is similar to COCs’, but the absence of oestrogens reduces control of the cycle, potentially causing spotting or irregular menses; moreover, ingestion must be regular to reduce the risk of unintended pregnancies (Pearl Index, referred to typical use, is 1–5, see Table 2).

POPs undergo first pass liver metabolism, potentially interacting with calcineurin inhibitors [82, 83].

Thanks to their safer cardiovascular profile, POPs are not contraindicated in dialysis patients; however, spotting may be bothersome in the case of heparin administration in dialysis. Conversely, since they reduce menstrual bleeding (or interrupt menses), they can be useful in patients with intense bleeding.

The thrombotic risk in patients taking a progestin-only contraceptive is debated. A Mexican trial [89] compared adverse effects in three group of 54 patients each, one group using COCs, one POPs, and one IUDs: four patients with systemic lupus erythematosus had thromboses while receiving hormones (two patients with COCs, two patients with POPs). On this basis, the contraceptive guidelines suggest caution in POP use in patients with systemic lupus erythematosus or nephrological complications: WHO puts progestin-only contraceptives in category 2 or 3. In recent years, the evidence has been reassuring, suggesting a low thrombosis risk, and this contraceptive is being more widely used. The RCOG guidelines now state that “The available evidence does not demonstrate an increased risk associated with POPs” [89–93].

A meta-analysis showed that the risk of thromboembolism in women using a low-dosage progestin-only contraceptive is comparable to that of non-users (RR 1.03, CI 95% 0.76–1.39 according to Mantha 2012 [94]) and does not increase even in women at high thromboembolic risk. Evidence on women with rheumatic diseases is lacking, and positions are different: WHO does not recommend use by patients with antiphospholipid antibodies (category 3), while RCOG considers progestin-only contraceptives a safe alternative to CHCs for patients with SLE, active nephritis and vascular diseases [90, 94].

In a recent review on contraception in patients with rheumatic diseases, Sammaritano concludes that progestin-only contraceptives are generally safe and that “the risks associated with any hormonal contraceptive method must be balanced with the risks of unintended pregnancy” [95].

Progestin-only subdermal implants (Etonogestrel or Levonorgestrel) are a fairly long-term (3 years), reversible and relatively safe method, with a failure rate of 0.1% (see Table 2). Their main adverse side effect is irregular menstrual bleeding or amenorrhea, as with POPs. WHO cautions against their use by patients with cardiovascular diseases or diabetes, mainly because of the risk of weight gain (although this is debated and weight gain is usually small). Implants can cause a reduction in bone mineral density, albeit less than those caused by other long-acting formulations (DMPA) [85, 96–102].

There are other injectable progestin-only therapies widely used as contraceptives. Two molecules are available: Depot-medroxy-progesterone acetate (DMPA) and Norethisterone Enanthate (NET-EN). Both highly effective, they differ in frequency of administration. Women taking DMPA are more likely to develop amenorrhoea, but there are no differences regarding other side effects. The injection of DMPA (150 mg) is used mostly in developing countries, because the injection is expensive (less than 1 dollar) and its effect lasts for 3 months. DMPA is available in Italy, but is only registered for use in treating endometrial and breast cancers. DMPA can reduce bone mineral density and this side effect can be relevant in patients on long-term steroid treatment, but the reduction tends to be reversible on discontinuation. Norethisterone Enanthate (NET-EN, dosage 200 mg), the other available injectable progestin-only contraceptive, has a duration of action of 2 months [103].

## Intrauterine devices (IUD)

- Medicated or non-medicated intrauterine devices are efficacious contraceptive alternatives in CKD patients (grade of evidence: high for the general population, low for the specific categories related to CKD patients).
- Intrauterine devices are associated with a slightly higher risk of pelvic infections in the first 20 days after placement, and their use is restricted in patients with malformations or pyelonephritis, and in those on peritoneal dialysis. Attention must be paid to the placement procedure (grade of evidence: high for the general population, low for the specific categories related to CKD patients).
- The main advantage of the non-medicated copper-only IUD is the avoidance of drug interactions; LNG-IUD has the advantage of reducing the amount of menstrual blood loss (grade of evidence: high for the general population, low for the specific categories related to CKD patients).
- Extrauterine pregnancies appear to be more frequent in women using IUDs (grade of evidence: high for the general population, low for the specific categories related to CKD patients).

IUDs (intrauterine devices, also called “coils”) are small, T-shaped, flexible devices that are inserted in the uterus; they stay in place for 3, 5 or 10 years, depending on the total surface occupied by the copper and on the dosage of the progestin released, but can easily be removed before term if complications arise or a woman wishes to conceive.

Two main types of IUDs are available: the copper-only IUD (5–10 years of efficacy, according to type), that do not contain any active drugs, and a medicated form, containing long-acting progesterone (Levonorgestrel, LNG-IUD, which has 3–5 years of efficacy).

The copper-only IUDs release copper ions, which are toxic to sperm; moreover, they induce local modifications of the endometrium and cervical mucus that prevent fertilization. The medicated forms release a small dose of Levonorgestrel (14–20 mcg/day) that thickens the cervical mucus, preventing the movement of spermatozoa. To a lesser degree, they can also impair ovulation. Their placement in primiparas and multiparas is easy because the cervix is already dilated, while in nulliparas it can be more complicated, in particular for the older forms; recent data show that the placement may be easy also in nulliparous women with a minimal discomfort [103]. Placement in patients with previous caesarean section requires caution (for example, ultrasound-guided placement). Placement can sometimes be painful, but the procedure is fast [104].  A copper-bearing IUD can also be inserted as an emergency contraceptive method within 120 h from unprotected sexual intercourse [105].

The IUD is a very effective method, with a low risk of failure (0.1–0.6%, Table 3). Since it is a long-term contraceptive method, compliance is very high. Its greatest advantage is probably that it makes it possible to avoid using active drugs, a safer choice in diseases associated with thromboembolic risk, risk of hypertension and weight gain.

According to WHO indications, the copper-bearing IUD can be safely used in patients with hypertension, diabetes with nephropathy, deep thromboembolism, and lupus. Antiphospholipid antibodies are instead a contraindication for the use of a medicated IUD [29].

The thrombotic risk of LNG-IUDs, given their progestin content, has been debated, but the evidence shows that the risk is low. A meta-analysis by Mantha et al. showed that the LNG-IUD was not associated with a higher thrombotic risk (RR 0.61, CI 95% 0.24–1.53) [94]. Sammaritano concluded that LNG-IUDs are safe and effective even in patients with rheumatic diseases [95]. According to EULAR (the European League Against Rheumatism), LNG-containing IUDs should be considered only if the benefits of the released hormone outweigh the risk of thrombosis”. RCOG states that there is little or no increased risk of VTE associated with the use of an LNG-IUD [94, 95, 106, 107].

IUDs are associated with a higher risk of extrauterine pregnancy, an event that, however, has a very low cumulative incidence and seems to have been associated with the first generation of IUDs. The risk is about 6 times higher than in the overall pregnant population and also exists in cases of previous use (OR 1.7% CI 95% 1.39–2.13, according to Li [108]) and above all in cases of IUD failure (OR 16.43%, 95% CI 10.42–25.89, according to Li [108]; 3.99 (95% CI 2.06–7.72 according to Gaskins [109]). Two rare adverse effects are perforation of the uterus, related to the insertion procedure, and expulsion of the device. Commonly, the copper-bearing IUD induces a small increase in duration of menstruation and blood loss, while the LNG-IUD often reduces the flow and sometimes induces amenorrhea, and may therefore be useful in patients with anaemia [108–114].

The risk of infection related to IUDs is a matter of ongoing debate. The timing of the increased risk is interesting: in a large retrospective study, cited in the RCOG guidelines, the overall risk of PID was 0.54% (95% CI 0.48–0.60) within 90 days after placement; according to a review of 13 trials, the risk of contracting a pelvic inflammatory disease is generally higher in the 20 days following IUD placement, suggesting that most infections are associated with the placement procedure. This means that many could therefore be avoided by a combination of pre-placement identification of infections (bacteriologic testing), providing treatment if infections are found, and careful asepsis and/or antibiotic prophylaxis. After this time, the risk remains low unless there is exposure to sexually-transmitted infections. It is not clear if the risk of infection is lower with LNG-IUDs than Cu-IUDs: one trial reported that the cumulative rate of PID was lower in women using LNG-IUDs compared to women using Cu-IUDs (cumulative discontinuation rates caused by PID for Cu-IUD 2.0 versus LNG-IUD 0.5, p < 0.013). However, in another study, PID rates did not differ between the two methods (termination rate because of PID: 0.7 for both Cu-IUDs and LNG-IUDs) [106, 115–118].

Regarding kidney transplantation, intrauterine devices are rated in category 3 for initiation of contraception, 2 for continuation [28]. Their placement in category 3 is probably related to occasional failure reports, but there is no evidence of an increase in risk of infections. IUDs are historically contraindicated in immunosuppressed women, since the IUD elicits a local inflammation that could be inhibited by immunosuppressive therapy. However, recent data do not confirm this finding, as they suggest that local inflammation induced by IUD involves macrophages, whereas iatrogenic immunosuppression involves lymphocytes; the few studies addressed to this question failed to find a significant difference in IUD failure between patients on immunosuppressive drugs and healthy women. There are no data regarding the risk of infection in transplanted patients, but no increase in the rate of infection has been found in immunocompromised HIV positive women using IUDs. It is against this background that the US Center for Disease Control and Prevention’s Medical Eligibility Criteria (CDC-MEC) for 2016 supports the use of the IUD only following uncomplicated kidney transplantation. The CDC MEC warns that the risks may outweigh the benefits for complicated transplants [28, 119, 120].

## Barrier methods

- Barrier methods include condoms, diaphragms, cervical caps and sponges. Their main drawback is their low efficacy, often linked to errors in use (grade of evidence: high for the general population, low for the specific categories related to CKD patients).
- The barrier methods that contemplate the use of a spermicide, such as diaphragms or cervical caps, can increase the risk of urinary tract infections and should be used with care in immunosuppressed patients and in patients with urinary tract infections and/or malformations (grade of evidence: high for the general population, low for the specific category related to CKD patients).
- The main advantage of using barrier methods is their lack of side effects (grade of evidence: high for the general population, low for the specific categories related to CKD patients).
- The condom (male or female) is the only contraceptive method that protects against sexually transmitted diseases (grade of evidence: high for the general population, low for the specific categories related to CKD patients).

Barrier methods are contraceptives that function by preventing direct contact between spermatozoa and an ovum. However, barrier methods are not as effective as other methods and cannot be considered an effective long-term contraceptive option, even if they are safe and do not interact with drugs. WHO does not pose any contraindications to their use [28].

The most widespread barrier method is the male condom, but other barrier methods exist: the diaphragm (plus spermicide), the cervical cap (plus spermicide), the cervical sponge (not available in Italy) and the female condom (Table 9). The failure rate depends largely on correct positioning of the device and on the method itself. Spermicide use increases the risk of urinary tract infections, in particular those caused by *Staphylococcus saprophyticus* and *E. coli*; the pathogenesis is not completely understood, but it seems that spermicides may alter the vaginal environment, leading to a greater colonization by uropathogens. Care must therefore be taken if they are used by immunosuppressed patients or by patients with frequent urinary tract infections or malformations [121–127].

**Table 9:** Failure rates, advantages and disadvantages of barrier methods. Adapted from [121]

Method	Image	Advantages	Disadvantages	Failure rate* (%)
Male condom		Little training needed; it is indicate for unexpected or occasional sex acts or in teenagers that are insecure about their bodies; inexpensive	Not reusable; it can reduce excitation and cause discomfort	2–15
Female condom		Latex free; more suitable when used with a lubricant	More complicated insertion than male condom; training is necessary	5–21
Diaphragm		Reusable; inexpensive	Does not protect against STDs; must be inserted before intercourse; training is necessary; discomfort during intercourse; not always suitable for multiparas or women with prolapse; not latex free; the use of spermicides can irritate the vagina and induce vaginal infections	6–16
Cervical Cap		Same as the diaphragm	Same as the diaphragm	6–16
Sponge		Same as the diaphragm	Same as the diaphragm, but contains spermicide	9–16

*STD* sexually transmitted disease

## Emergency contraception

- Emergency contraception is based on a high dosage of progestin or placement of a copper-bearing IUD. The use of emergency contraception should be considered within 120 h after unprotected sexual intercourse (grade of evidence: high for the general population, low for the specific categories related to CKD patients).
- The main drawbacks are the risk of nausea and vomiting and potential interaction with calcineurin inhibitors (grade of evidence: high for the general population, low for the specific categories related to CKD patients).

Emergency contraception is the term used for forms of contraception that are effective in preventing an unintended pregnancy when administered within a specified period of time after unprotected, or inefficaciously protected sexual intercourse. Emergency contraception can be achieved by administering orally a high dose of progestin or the selective progestin receptor modulator (in Italy the available formulations are a single dose of 30 mg of Ulipristal acetate or a single dose of 1.5 mg of Levonorgestrel) or by placing an IUD within 5 days after unprotected intercourse; due to their lower cost, copper-bearing IUDs are more often employed. Their efficacy decreases as time passes and is variable: the efficacy of progestin ranges from 54 to 99% within the first 72 h; Ulipristal acetate is three times more effective than Levonorgestrel. The IUD has an efficacy of 99% when placed within 120 h after unprotected intercourse [128–132].

The mechanism of action of high-dose progestin consists in thickening the cervical mucus and interfering with ovulation: before the peak of LH (luteinizing hormone) they inhibit follicular maturation and prevent ovulation; UPA inhibits ovulation even after the onset of the LH peak, delaying ovulation for at least 5 days. The side effects of high-dose progestin are not different from those reported for POPs and other progestin formulations, although vomiting is more frequent. In case of vomiting within 2 h, the dose should be repeated, but the systematic use of anti-emetic drugs is not recommended. In spite of the high dosage of progesterone, this contraceptive does not contain oestrogens and can be considered reasonably safe in patients with kidney disease; however, it is not recommended in severe chronic liver disease. WHO does not warn against its use in any category, including the presence of deep venous thrombosis. Frequent use of EC in women with MEC category 2, 3 or 4 has to be discouraged; furthermore, a high dose of a steroid contraceptive can interact with the metabolisms of several drugs, including calcineurin inhibitors (see section on combined hormonal contraceptives). The oral emergency contraceptives do not prevent the implantation of a fertilized egg and do not affect an existing pregnancy. After taking an emergency contraceptive, patients often have menstrual irregularities [30, 128–132].

## Surgical sterilization

- Surgical sterilization is the only non-reversible contraceptive method (grade of evidence: high for the general population, no data for the specific categories related to CKD patients).
- The main risks are those linked with the surgical procedure (grade of evidence: high for the general population, no data for the specific categories related to CKD patients).

Surgical sterilization is the only non-reversible contraceptive method; it includes female surgical sterilization (tubal sterilization) and male surgical sterilisation (vasectomy). While it is the method least frequently used in Europe, sterilization is the contraceptive method most used by women elsewhere in the world, particularly women in China, India and the United States. Before 1978, surgical sterilization was illegal in Italy (Article 552/1930 of the Italian Criminal Code); since 1978, (Law 194/78. Article 22), an adult woman has been allowed to ask for tubal sterilization if the procedure is expected to provide a health or psychological, benefit. In 2018 6% of married women in Italy chose this method (Table 3) [30].

Female sterilization (salpingectomy) consists in the removal of the fallopian tubes or in their ligature (occlusion), and can be performed by laparoscopy or by laparotomy. The first procedure involves ligature of the tubes and removal of a small section; because of the possibility of spontaneous reopening of the tubes and failure of the procedure, even if correctly performed, and because of the evidence that ovarian cancer often originates in the fimbriae of the fallopian tubes, the surgical technique generally used nowadays consists in the removal of the tubes in their entirety, followed by histological analysis. Sometimes, generally during caesarean section, the removal of the tubes is technically difficult or not possible and in these cases occlusion is preferred.

WHO recommends caution in patients with kidney diseases. In fact, sterilization bears the risks of infection and haemorrhage, not specifically linked to the procedure itself, but common in all types of abdominal surgery [28].

## Abortion

- Abortion should not be considered a birth control procedure, and should not be used as such (grade of evidence: high for the general population, no data for the specific categories related to CKD patients).
- Surgical abortion may impair subsequent fertility and involves the risks common to all surgical procedures (grade of evidence: high for the general population, no data for the specific categories related to CKD patients).
- There is limited evidence regarding the use of “medical”, drug-induced abortion in CKD patients, and although it is less invasive, the procedure cannot be considered to be safe for CKD patients (grade of evidence: no data for the specific categories related to CKD patients).

Abortion is defined as the spontaneous or provoked interruption of a pregnancy before the 180th day (23rd week) of gestation. According to this definition, abortion cannot be considered a contraceptive method, since conception has already occurred. We have included it in this review because it is an important item in reproductive health. Elective abortion can be voluntary, when requested by a pregnant woman, or therapeutic, when indicated for clinical or psychological reasons. Abortion is a controversial question throughout the world and laws regulating voluntary termination of pregnancy vary between countries. In some developing countries abortion is illegal but unofficially practiced, with high risks for women’s health and lives. In Europe, abortion is illegal in Malta, San Marino and Vatican City. In Italy a 1978 law (194/1978) legalized voluntary abortion and since then abortion without medical indication has been legal in the first 90 days of pregnancy, while abortion for therapeutic reasons can be practiced after the 90th day if the pregnancy represents a serious hazard for the woman’s life or well-being [133–135].

The procedure can be medical or surgical.

The drugs used for medical termination of pregnancy are Mifepristone and Misoprostol. Limited data are available on the use of these drugs in patients with CKD. Mifepristone is metabolized by the liver, leading to potential drug interactions, while Misoprostol is excreted by the kidneys. In patients with reduced clearance, this can lead to increased bioavailability and higher peak concentrations of Misoprostol. There is not yet enough data available to show that reducing doses is advisable, and clinical surveillance is needed in patients with severe CKD. Animal studies have shown a worsening in kidney function after Mifepristone, but data on CKD women are not available. We were able to find only one published case series of three women with CKD (stage 4 or 5) which reported no complications after taking medical abortifacients [136, 137]. Medication may be preferable to a surgical procedure (vacuum aspiration and/or curettage) for women who wish to become pregnant again, because interventions on the uterus can impair subsequent placentation, especially if repeated. A Cochrane meta-analysis has shown that a medical procedure in the first trimester of pregnancy is related to a longer duration of bleeding than a surgical procedure (OR 2.94, 95% CI 2.10–3.78), but the difference in total blood loss is not significant (OR 1.90, 95% CI 0.05–3.75); this could be relevant in patients with anaemia [137, 138]. Surgical procedures are considered to low complexity ones, but once more data regarding the preferred procedure in patients with CKD are not available.

## Summary remarks

Pregnancy is a challenge in CKD, but its improved probability of success makes contraception another important challenge for CKD patients.

In the early CKD stages (stages 1–2–3a), in patients without hypertension or proteinuria (except in SLE + aPL), virtually all options are available. Particular care should be paid to an increase in or development of proteinuria in predisposed patients and to the development of urinary tract infections, in particular when patients use spermicide or a vaginal ring.

The contraceptive pill should be avoided in every CKD stage in hypertensive patients and in patients in stages 3b–5, as well as in patients with pro-coagulatory status, including systemic diseases such as SLE, or nephrotic proteinuria. The options in these cases include progestin-only contraceptives, which can, however, cause spotting (sometimes increased by heparin use in dialysis patients), intrauterine devices, barrier methods and surgical sterilization.

As previously mentioned, the use of spermicides and the vaginal ring should be prescribed and monitored with care in patients with recurrent urinary tract infections, while data on intrauterine devices suggest that the risk of pelvic infection is slightly higher after insertion; infection can be avoided by previous targeted treatment, ensuring aseptic insertion, and possible antibiotic prophylaxis.

Dialysis patients are not good candidates for the pill, and alternative solutions should be sought; given their reduced fertility, barrier methods or non-medicated intrauterine devices are possible alternatives, while no contraindications, besides spotting, exist for progestin-only contraceptive agents.

In kidney transplant patients the use of the pill should be limited to the few patients with normal kidney function, normotension and no proteinuria, while alternative solutions need to be sought in all the other cases. Barrier methods are limited by their lower efficacy, except for ideal use, and in the case of choice of non-medicated intrauterine devices, attention to insertion procedures is warranted. No contraindications, except for spotting, exist for progestin-only birth control agents; however, due to potential pharmacologic interactions, the level of antirejection drugs (in particular calcineurin inhibitors, but also all the drugs metabolised in the cytochrome P450 pathway), should be carefully monitored.

While no formal contraindications exist for emergency progestin use, this treatment should not be routinely used in patients with occasional intercourse, and the risk of pharmacologic interactions should be borne in mind (in particular with calcineurin inhibitors).

**Fig. 1:**
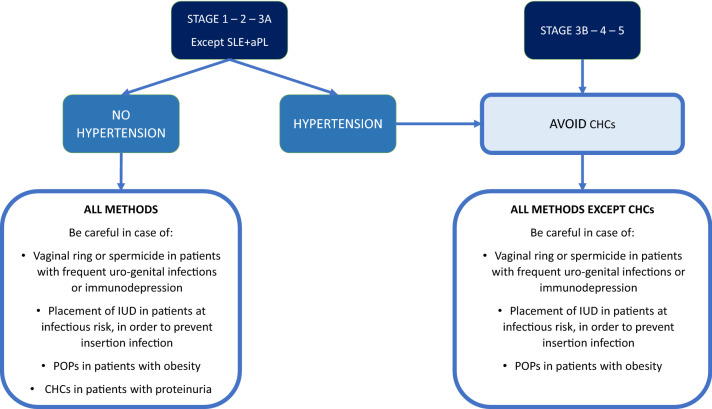
Flow chart on contraception in CKD patients. *SLE* systemic lupus erythematosus, *aPL* antiphospholipid antibodies, IUD intrauterine device, *POP* progestin-only pill, *CHC* combined hormonal contraceptive, *CKD* chronic kidney disease

Abortion is not, and should never be used as a means of birth control. It should be avoided whenever possible in all CKD women, in whom the physical and psychological balance between low fertility and unwanted pregnancy is particularly difficult. When a pregnancy needs to be interrupted, the limited evidence available on the lack of side effects of “medical” abortion should be balanced against the potential advantage of avoiding invasive procedures that risk further reducing fertility. These challenges are summarized in Fig. 1, depicting a treatment flow-chart based on the current knowledge on birth control in CKD patients.
